# Supplementary material for: Electronic ferroelectricity in monolayer graphene moiré superlattices
Source: Nat Commun. 2024 Dec 30;15:10905. doi: 10.1038/s41467-024-55281-z (PMC11685881; doi:10.1038/s41467-024-55281-z)
Supplement: Supplementary file 1 — Supplementary Information [file 41467_2024_55281_MOESM1_ESM.pdf]

# Supplementary Information for

# Electronic ferroelectricity in monolayer graphene moiré superlattices

Le Zhang[1,2], Jing Ding[1,2], Hanxiao Xiang[1,2], Naitian Liu[1,2], Wenqiang Zhou[1,2], Linfeng Wu[1,2], Na Xin[3*], Kenji Watanabe[4], Takashi Taniguchi[5], Shuigang Xu[1,2*]

[1] *Key Laboratory for Quantum Materials of Zhejiang Province, Department of Physics, School of Science, Westlake University, 18 Shilongshan Road, Hangzhou 310024, Zhejiang Province, China*

[2] *Institute of Natural Sciences, Westlake Institute for Advanced Study, 18 Shilongshan Road, Hangzhou 310024, Zhejiang Province, China*

[3] *Department of Chemistry, Zhejiang University, 310058, Hangzhou, China*

[4] *Research Center for Electronic and Optical Materials, National Institute for Materials Science, 1-1 Namiki, Tsukuba 305-0044, Japan*

[5] *Research Center for Materials Nanoarchitectonics, National Institute for Materials Science, 1-1 Namiki, Tsukuba 305-0044, Japan*

[*]Corresponding authors. Email: na.xin@zju.edu.cn (N.X.), xushuigang@westlake.edu.cn (S.G.X.)

## Contents

**Supplementary Text**

Supplementary Note 1. Distinguishing monolayer from bilayer graphene

Supplementary Note 2. Crystallographic alignment and twist angle determination

Supplementary Note 3. Excluding extrinsic effects that account for the hysteresis

Supplementary Note 4. Scan-range dependent GSAS and hysteresis

Supplementary Note 5. Detailed data from Hall measurement

Supplementary Note 6. Characterization of additional devices

**Supplementary Fig. 1 to 21**

**Supplementary Table 1**

**Supplementary References**

**Supplementary Note 1: Distinguishing monolayer from bilayer graphene**

Although unconventional ferroelectricity has been observed in Bernal bilayer graphene/h-BN superlattice, its underlying mechanism is still mysterious. Previous understanding is that layer-polarized flat moiré bands and interlayer charge transfer between top and bottom graphene layers play an important role[1,2].

Firstly, the layer number of graphene can be determined via reflection contrast spectroscopy. As depicted in Supplementary Fig. 1b and 1c, the exfoliated graphene flake shows consecutive steps. The optical contrast relative to the 285 nm $SiO_2$/Si substrate decreases with the increasing graphene layer number monotonously, following the Beer-Lambert law. The layer-number-dependent optical contrast helps us distinguish the monolayer from the bilayer and tri-layer graphene. Our transport measurements from monolayer, bilayer, and trilayer graphene were performed from the devices sharing the same stack, which allows us to *in-situ* compare their characteristics.

Secondly, Raman spectra are capable of determining layer number of graphene for layer thickness of less than four layers[3]. The 2D peaks of graphene in Raman spectra are sensitive to the layer number. As shown in Supplementary Fig. 1f, the 2D peak from monolayer graphene is sharper and exhibits slightly redshift, compared with bilayer graphene. Moreover, it can be fitted by a single Lorentz function with a full width at half maximum (FWHM) of 32 $cm^{-1}$, which is a typical feature of single-aligned monolayer graphene/h-BN stack[4].

Thirdly, the transport behaviors of monolayer graphene and bilayer graphene moiré superlattice exhibit distinguishable features. Generally, the peak resistance of SDP at hole side is comparable with that of CNP and SDP shows prominent electron-hole asymmetry in monolayer graphene/h-BN superlattice[5-8]. By contrast, bilayer graphene/h-BN superlattice exhibits weak hole-side SDP[9]. This gives us an empirical method of distinguishing monolayer and bilayer graphene directly from transport measurements (see Fig. 1b-c).

Fourthly, in bilayer graphene, the external electric field can open a gap at CNP with its size increases with increasing electric field. By contrast, the CNP of monolayer graphene is almost resilience to the electric field. We have measured the dual-gate dependent $R_{xx}$ near CNP for monolayer and bilayer graphene, shown in Supplementary Fig. 2. As expected, the CNP peak increases dramatically with electric field in bilayer graphene, while remaining in the same order of magnitude in monolayer counterpart.

Overall, we can unambiguously confirm that the measurements in the main text are from monolayer graphene/h-BN. Therefore, we conclude the unconventional ferroelectricity can emerge in monolayer graphene/h-BN superlattice.

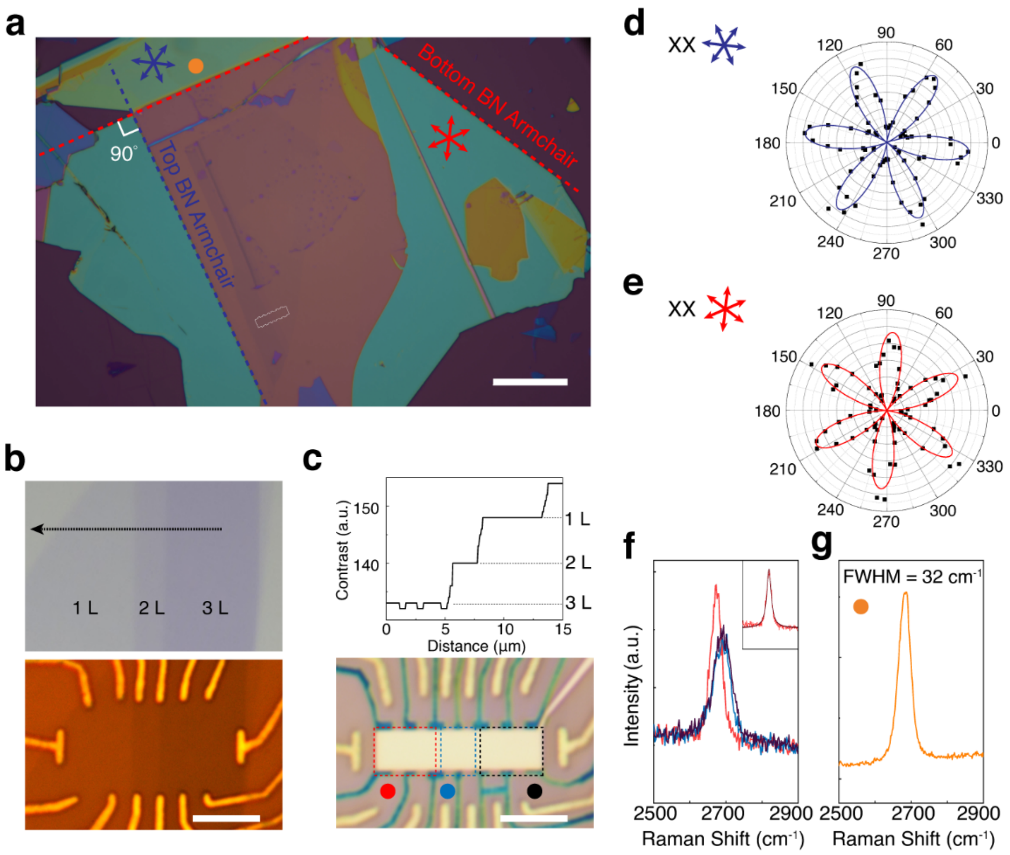


**Supplementary Fig. 1 | Optical characterizations of Device D1. a**, Optical image of the h-BN/graphene/h-BN heterostructure. The bule (red) dashed lines correspond to the armchair crystallographic axes of top (bottom) h-BN. The white dashed lines indicate the Hall bar configuration. The scale bar is 10 μm. **b**, Optical image of graphene flake before encapsulation and after nanofabrication, in which the mono-/bi-/tri-layer are labelled. The scale bar is 5 μm. **c**, Cross-sectional profile of optical contrast along the black dashed arrow in (**b**). Bottom panel: optical image of final Hall bar device, where the red/bule/black regions are mono-/bi-/tri-layer graphene channels, respectively. The scale bar is 5 μm. **d**,**e**, SHG signals for top and bottom h-BN, respectively. The relative angle between them is ~30°. **f**, Comparison of Raman spectra acquired in mono-/bi-/tri-layer graphene, marked as red/bule/black spots in (**c**). The inset shows the single-peak Lorentz fitting of Raman signal in monolayer graphene. **g**, Raman spectrum of monolayer graphene, yielding a full width at half maxima (FWHM) value of 32 $cm^{-1}$. The data were acquired at the orange spot in (**a**), where it's solely covered by top h-BN. With this, we can identify that graphene is singly aligned with top h-BN.

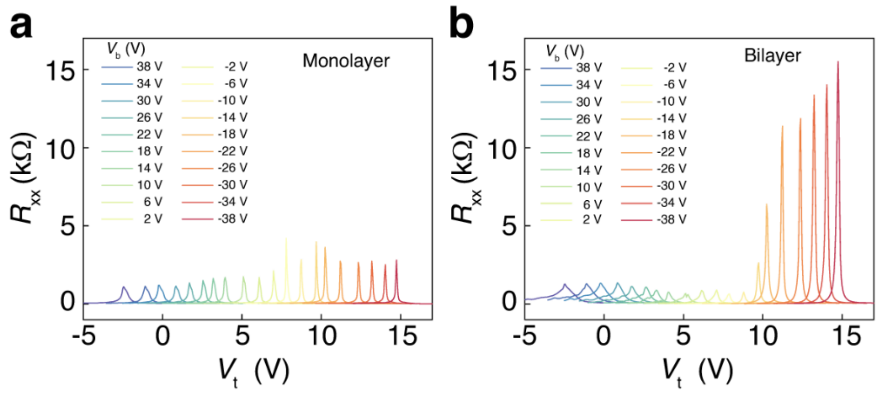


**Supplementary Fig. 2 | Field-dependent longitudinal resistance near CNP of mono- and bi-layer graphene. a**,**b**, Longitudinal resistance of monolayer (**a**) and bilayer (**b**) graphene as a function of $V_\mathrm{t}$ at fixed $V_\mathrm{b}$ ranging from -38 V to 38 V. Data were collected by fixing $V_\mathrm{b}$ and scanning $V_\mathrm{t}$ from -15 V to 15 V. For better comparison, we only plotted the resistance peaks near CNP and kept them in the same plotting scale. Due to the screening effect, the resistance peaks of bilayer graphene are almost the same when $V_\mathrm{b}$ decrease from 38 V to 2 V. When $V_\mathrm{b}$ decrease further from -2 V to -38 V, the resistance peaks increase dramatically. In monolayer graphene, the resistance peaks of CNP remain in the same order of magnitude within the whole scanning range.

**Supplementary Note 2: Crystallographic Alignment and twist angle determination**

We utilized Raman spectra and SHG to figure out the alignment between graphene and h-BN. During the van der Waals assembly process, we intentionally aligned graphene with both top and bottom h-BN by using their straight edges. The result can be either single alignment or doube alignement. The Raman spectra of monolayer part in final stack yields a 2D peak with FWHM of 32 cm$^{-1}$ (Supplementary Fig. 1f), indicating the single alignment[4]. We further confirmed the top h-BN and bottom h-BN have a relative angle of 30° by SHG measurements as shown in Supplementary Fig. 1d and 1e.

To determine whether the top h-BN or bottom h-BN is aligned with graphene, we intentionally reserved one part of monolayer graphene covered solely by top h-BN without any contact with bottom h-BN, as shown in Supplementary Fig. 1a. Our Raman spectra measured from this part shows a 2D peak with FWHM of 32 cm$^{-1}$ (Supplementary Fig. 1g), which is a typical feature of graphene/h-BN superlattice[4]. For reference, typcial non-aligned graphene has a 2D peak with FWHM of 20 cm$^{-1}$ [4]. With this, we can confirm that graphene was singly aligned with the top h-BN for Device D1.

To precisely determine the twist angle between graphene and top h-BN, we directly resort to the transport results. The moiré wavelength of moiré superlattice between graphene and h-BN is determined by $\lambda = \frac{(1+\delta)a_G}{\sqrt{2(1+\delta)(1-\cos\theta)+\delta^2}}$, where $a_G = 0.246$ nm is the in-plane lattice constant of graphite, $\delta \approx 1.6\%$ is the lattice mismatch between graphene and h-BN, and $\theta$ is the relative misalignment angle between the two lattices. From the transport measurement, we found the full filling carrier density of moiré superlattice in Device D1 was $n_\mathrm{Full} = 3.0 \times 10^{12}$ cm$^{-2}$, determined from the difference between hole-side SDP and CNP at normal regime without screening effect as

shown in Fig. 2d and 2h. The area of a moiré unit cell is $A = 4/n_{\text{Full}}$. Then we can obtain the moiré wavelength is 12.4 nm, by using $A = \frac{\sqrt{3}}{2}\lambda^2$. The twist angle between graphene and top h-BN is determined to be 0.68°.

**Supplementary Note 3: Excluding extrinsic effects that account for the hysteresis**

Gate hysteresis arising from extrinsic effects, such as charge traps induced by defects, impurities or absorbates, strongly depends on the scanning parameters of the gate[10,11]. To exclude these scenarios, we performed temperature-, scanning rate- and cycle number-dependent experiments.

1. In extrinsic effect (such as charge traps) dominant mechanisms, as schematized in Supplementary Fig. 3a, the hysteresis is highly dependent on the scanning rate, which is due to the existence of mobile ions or dipole moment in the system. In contrast, in our graphene superlattice devices, the measurements of longitudinal $R_{xx}$ by forward and backward sweeping $V_{\text{b}}$ at fixed $V_{\text{t}} = 0$ with different scanning rate demonstrate that the hysteresis is independent of the scanning rate in terms of the resistance peak position.

2. In Supplementary Fig. 4, we also examine the endurance of the hysteresis by repetitively scanning $V_{\text{b}}$ or $V_{\text{t}}$ forward and backward for 20 cycles. All the curves overlap with each other, demonstrating their good cycle endurance. We observed similar robust hysteresis and good endurance in monolayer, bilayer and tri-layer graphene moiré superlattice, indicating they share the common origins. We further performed the endurance measurements by switching the polarization states more than $10^4$ times (limited by the instrument) as shown in Supplementary Fig. 5. It's noted that these data were acquired six months after the initial measurements. Typically, charge traps will lead to performance degradation over several cycles. In contrast, our device does not show a substantial fatigue effect after $3 \times 10^4$ switching cycles.

3. In the main text, we briefly excluded the extrinsic mechanisms by temperature-dependent $P_{\text{2D}} - E$ hysteresis loops. In this section, we will elaborate on this. As illustrated in Supplementary Fig. 6a, gate hysteresis induced by extrinsic effects should exhibit an enhanced performance at high temperature because the charges acquire high energy to migrate. However, this is not the case in our system. We have measured longitudinal $R_{xx}$ by forward and backward sweeping $V_{\text{t}}$ at 2.2 K, 100 K and 200 K while fixing $V_{\text{b}} = 0$ in our monolayer graphene superlattice device, which shows a reverse behavior. The hysteresis tends to be weaker at the higher temperature. Such a behavior can also be manifested by both dual-gate mappings at elevated temperature (Supplementary Fig. 7) and thermal-activated remanent polarization $P_{\text{r}}$ at $|E|_{\text{max}} = 80$ mV nm$^{-1}$ (Supplementary Fig. 8a). In Supplementary Fig. 7, the GSAS regions (marked by double-headed arrow) shrink gradually with increasing temperature, showing the behavior opposite to charge trapping effects. The saturation itinerant density ($n_{\text{H}}^{\text{s}}$) is replotted as a function of reciprocal temperature by using an Arrhenius plot as shown in Supplementary Fig.8a, yielding an energy gap of ~367 K. These results demonstrate that part of localized carriers have been thermally excited from the moiré trapping potential at the elevated temperature, thus behaving itinerantly in the conducting channel.

The nonmonotonic evolution of $P_{\text{r}}$ with temperature at $|E|_{\text{max}} = 36$ mV nm$^{-1}$ provides additional evidence for the underlay mechanism described in Fig. 3b and 3e in the main text. When $|E|_{\text{max}} < 73$ mV nm$^{-1}$, the insufficient supply of localized carrier in Process i resulting in $n_{\text{H}}^{r} < n_{\text{H}}^{s}$

(or equivalent to $P_r < P_s$) at the base temperature. Meanwhile, one key feature of the $P_{2D} - E$ hysteresis loops is the slope (color dashed lines in Supplementary Fig. 8b) of the parallelogram loops is a constant, independence of the temperature, which is determined by the capacitance of the gate dielectric (h-BN). The moiré trapping effect becomes weaker at higher temperature, leading to a smaller saturation polarization $P_s$ value. To keep the slope unchanged, the windows of parallelogram loop need to be larger. In this scenario, taking $|E|_{max} = 36$ mV nm$^{-1}$ as an example, when increasing the temperature from $T = 2.2$ K to $T \sim 240$ K, although $P_s$ is decreasing, $P_r$ ($< P_s$) is increasing. At $T \sim 240$ K, $P_r = P_s$ is reached. Further increasing temperature from 240 K to 300 K, since the relationship of $P_r = P_s$ is unchanged, $P_r$ accompanied by $P_s$ is decreasing.

All the above features manifest that the hysteresis loops observed in graphene moiré superlattices are the intrinsic behavior arising from the moiré trapping effects, rather than the extrinsic mechanisms like charge trapping.

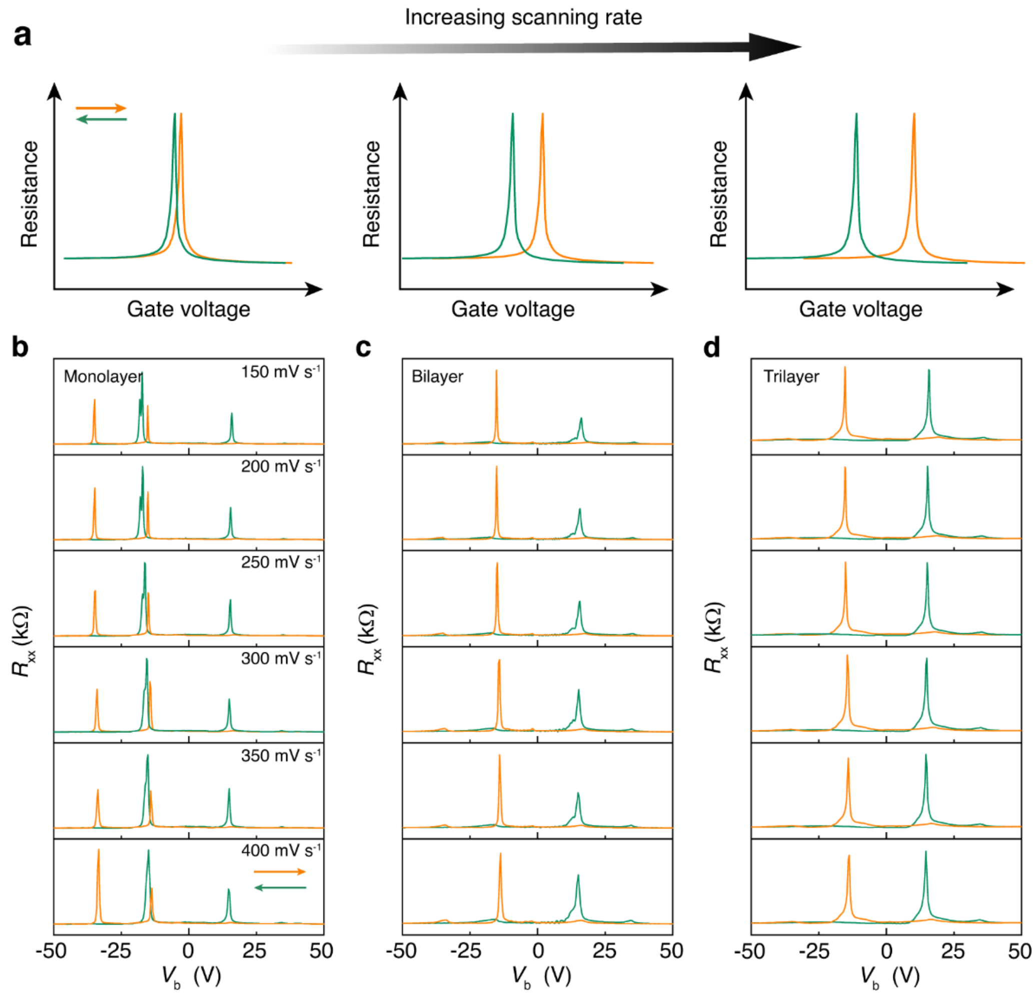


**Supplementary Fig. 3 | Independence of hysteresis on the scan rates of $V_b$. a,** Schematic of charge-trapping hysteresis in a typical graphene device. From left to right, the hysteresis become more pronounced with increasing scanning rate. **b-d**, Forward (orange) and backward (green) scans of $V_b$ for different scan rates in monolayer (**a**), bilayer (**b**) and tri-layer (**c**) graphene at a fixed $V_t =$ 0 V. From upper panel to bottom panel, no noticeable variation was observed in terms of the position and magnitude of resistance peaks with the scan rates increasing from 150 mV s$^{-1}$ to 400 mV s$^{-1}$.

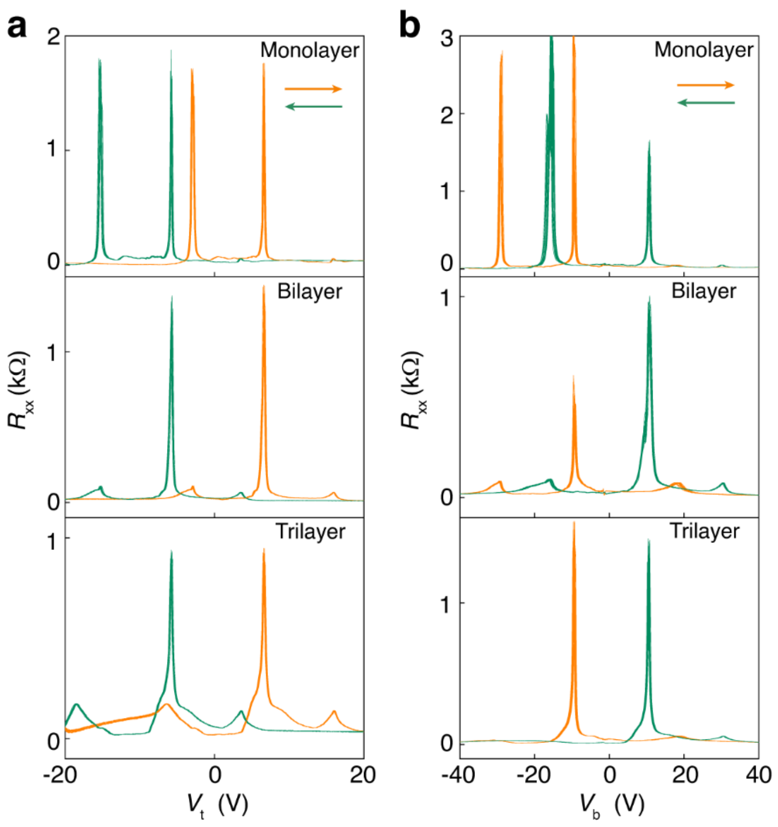


**Supplementary Fig. 4 | Robustness of hysteresis. a**,**b** Repetitive scan $V_t$ at a fixed $V_b = 0$ V (**a**) or scan $V_b$ at a fixed $V_t = 0$ V (**b**) for 20 cycles in mono-/bi-/tri-layer graphene. All the curves overlap with each other, manifesting that the hysteresis behaviors are robust.

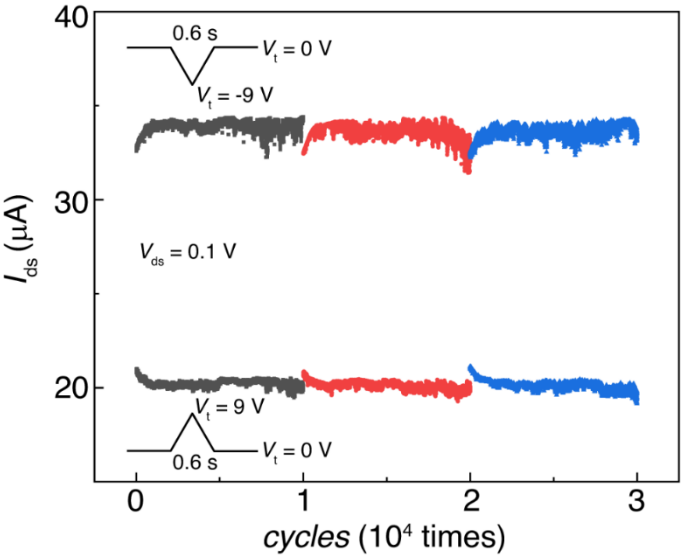


**Supplementary Fig. 5 | Endurance of graphene electronic ferroelectricity against switching cycles.** Channel current $I_{ds}$ of graphene moiré device measured with constant source-drain voltage $V_{ds}$ after a series of positive and negative pulse voltages applied on the top gate. For each cycle, we applied ± 9 V pulse with a width of 0.6 s. After $10^4$ cycles (the maximum cycles allowed in our equipment), we repeated them for another 2 rounds. Within the whole test process, the polarization states were highly stable.

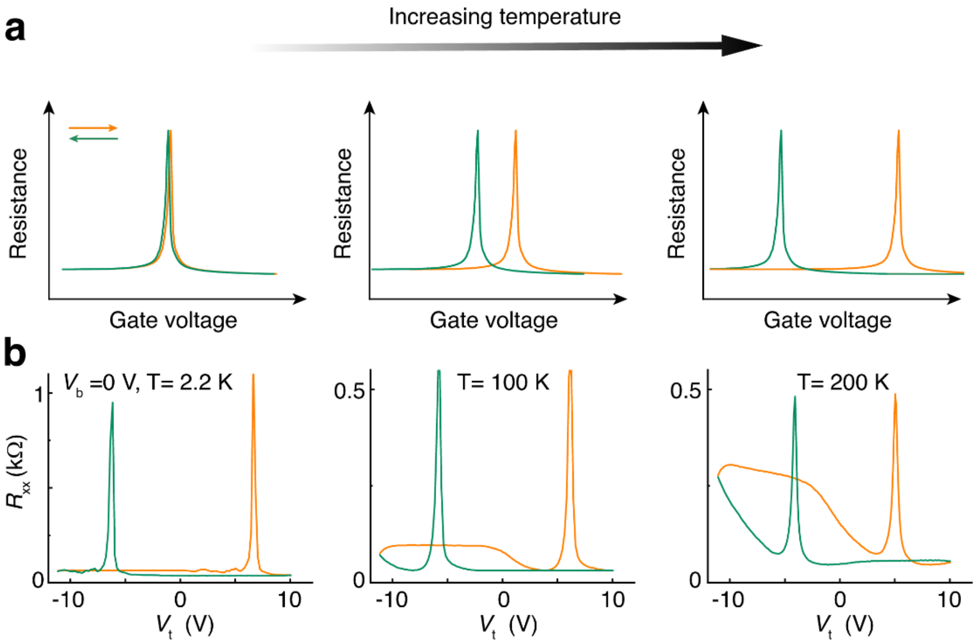


**Supplementary Fig. 6 | Temperature dependence of hysteresis. a,** Schematic of charge-trapping hysteresis in a graphene device. From left to right, hysteresis becomes more pronounced with increasing temperature. For the actual experiment, please refer to ref [10]. **b,** $R_{xx}$ as a function of $V_\mathrm{t}$ at 2.2 K, 100 K and 200 K while fixing $V_\mathrm{b} = 0$ in our monolayer graphene superlattice device.

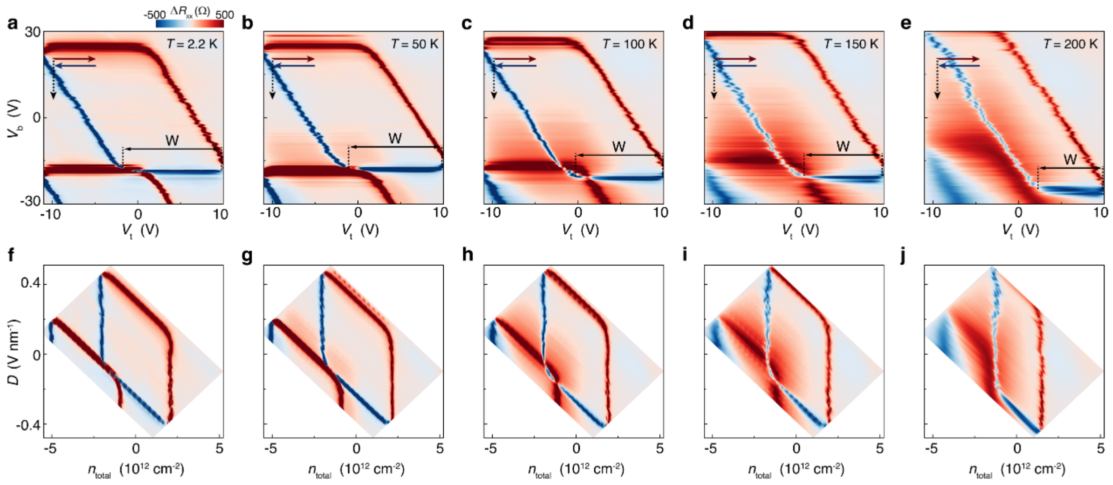


**Supplementary Fig. 7 | Temperature dependence of dual-gate mappings. a-e,** Difference in $R_{xx}$ between forward and backward scan as a function of $V_\mathrm{t}$ and $V_\mathrm{b}$ in monolayer graphene acquired at 2.2 K (**a**), 50 K (**b**), 100 K (**c**), 150 K (**d**) and 200 K (**e**). The fast-scan axis is $V_\mathrm{t}$, and the slow-scan axis is $V_\mathrm{b}$. **f-j** The corresponding resistance maps as a function of $n_\mathrm{total}$ and $D$ for (**a**)-(**e**), respectively. The width ($W$) of the GSAS regime and ferroelectric hysteresis gradually shrink with the increasing of temperature due to the thermal activation of localized carriers trapped by the moiré potential.

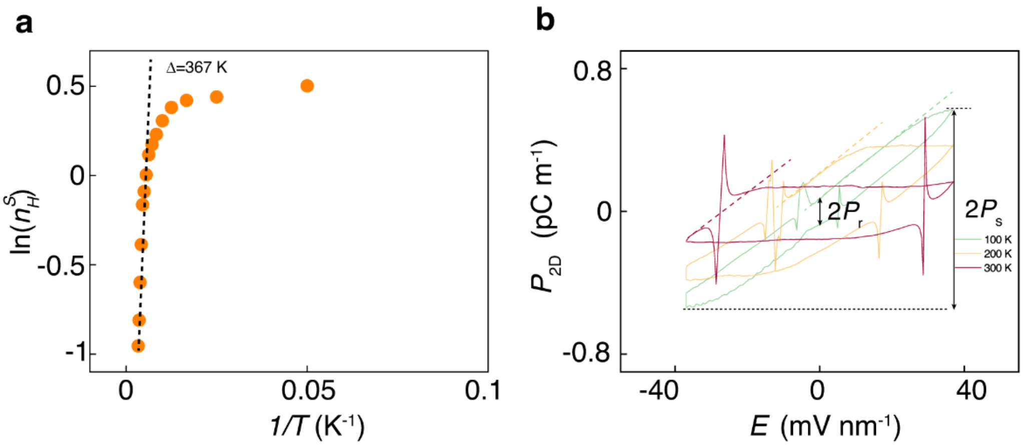


**Supplementary Fig. 8 | Thermal-activated behavior of $\mathbf{P_{2D} - E}$ hysteresis loops. a,** Arrhenius plot of saturation itinerant density $n_{\mathrm{H}}^{\mathrm{s}}$ as a function of temperature. Data were extracted from Fig. 4a in the main text. **b**, Replotting $P_{2D} - E$ hysteresis loops measured at 100 K, 200 K and 300 K for $|E|_{\max} = 36$ mV nm$^{-1}$. The slopes of each individual parallelogram loop are labeled as color dashed lines, corresponding to GSAS regions.

### Supplementary Note 4: Scan-range dependent GSAS and hysteresis

Aforementioned in Fig. 2 of the main text, $V_{\mathrm{t}}$ is screened in certain regions when measuring the dual-gate mappings of $R_{xx}$. Here, we provide a comprehensive explanation based on electronic ferroelectricity. As shown in Supplementary Fig. 9b, $V_{\mathrm{t}}$ is screened from -15 V to -3 V when scanning forward (marked by double-headed arrow), while screened from 15 V to 3 V when scanning backward. Based on the GSAS mechanism, such an anomalous screening can be unveiled as follows. For instance, let's consider the process of $V_{\mathrm{t}}$ sweeping from -15 V to 15 V at fixed $V_b = 0$ V. The initial state at $V_{\mathrm{t}} = -15$ V is composed of both itinerant holes and localized holes, which are injected in the last scan step. When $V_{\mathrm{t}}$ sweeping from -15 V to -3 V, $V_{\mathrm{t}}$ firstly extracts localized holes and then injects localized electrons till the half filling of a moiré band, thus making no contributions to the conducting channel. This process is GSAS, manifesting as horizontal lines as shown in Supplementary Fig.9b. When further increasing from -3 V to 15 V, $V_{\mathrm{t}}$ firstly neutralizes itinerant holes and then injects itinerant electrons. This process exhibits as normal gating effect, resulting in diagonal lines in CNP and electron-side SDP.

We note that GSAS is dependent on the scan range of the fast-scan axis. When the scan range of $V_{\mathrm{t}}$ is reduced, the parallelogram loops shrink gradually and eventually smear out when the scan range is smaller than a critical $V_{\mathrm{t}}$ (see Supplementary Fig. 9d). Similar behavior with weaker screening effect can be observed while sweeping $V_b$ forward and backward at fixed each $V_{\mathrm{t}}$ (Supplementary Fig.10). It's also noted that the anomalous screening and hysteresis are independent of the directions of slow-scan axis (see Supplementary Fig.11).

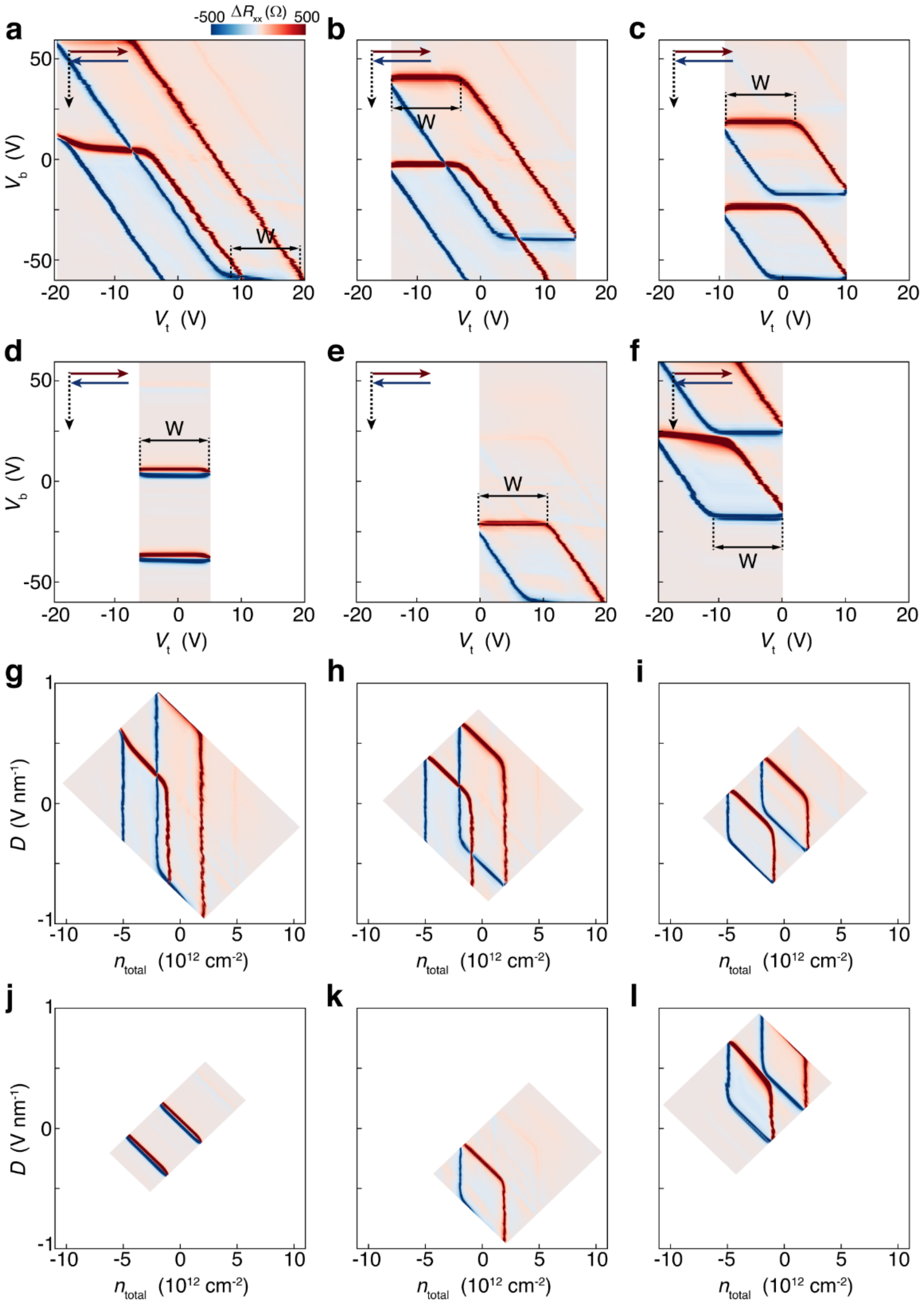


**Supplementary Fig. 9 | Dependence of ferroelectric hysteresis on the scan range of $V_t$. a-f**, Difference in $R_{xx}$ between forward and backward scan as a function of $V_b$ and $V_t$. The solid (dashed) arrows illustrate the fast (slow)-scan direction. The scan ranges of $V_t$ are ±20 V (**a**), ±15 V (**b**), ±10 V (**c**), ±5 V (**d**), 0 V to 20 V (**e**) and -20 V to 0 V (**f**). The width ($W$) of the GSAS regime corresponds to the screening capability of $V_t$. **g-l**, Replotted corresponding maps of resistance as a function of $n_{total}$ and $D$. Data were acquired at 2.2 K and plotted in the same scale.

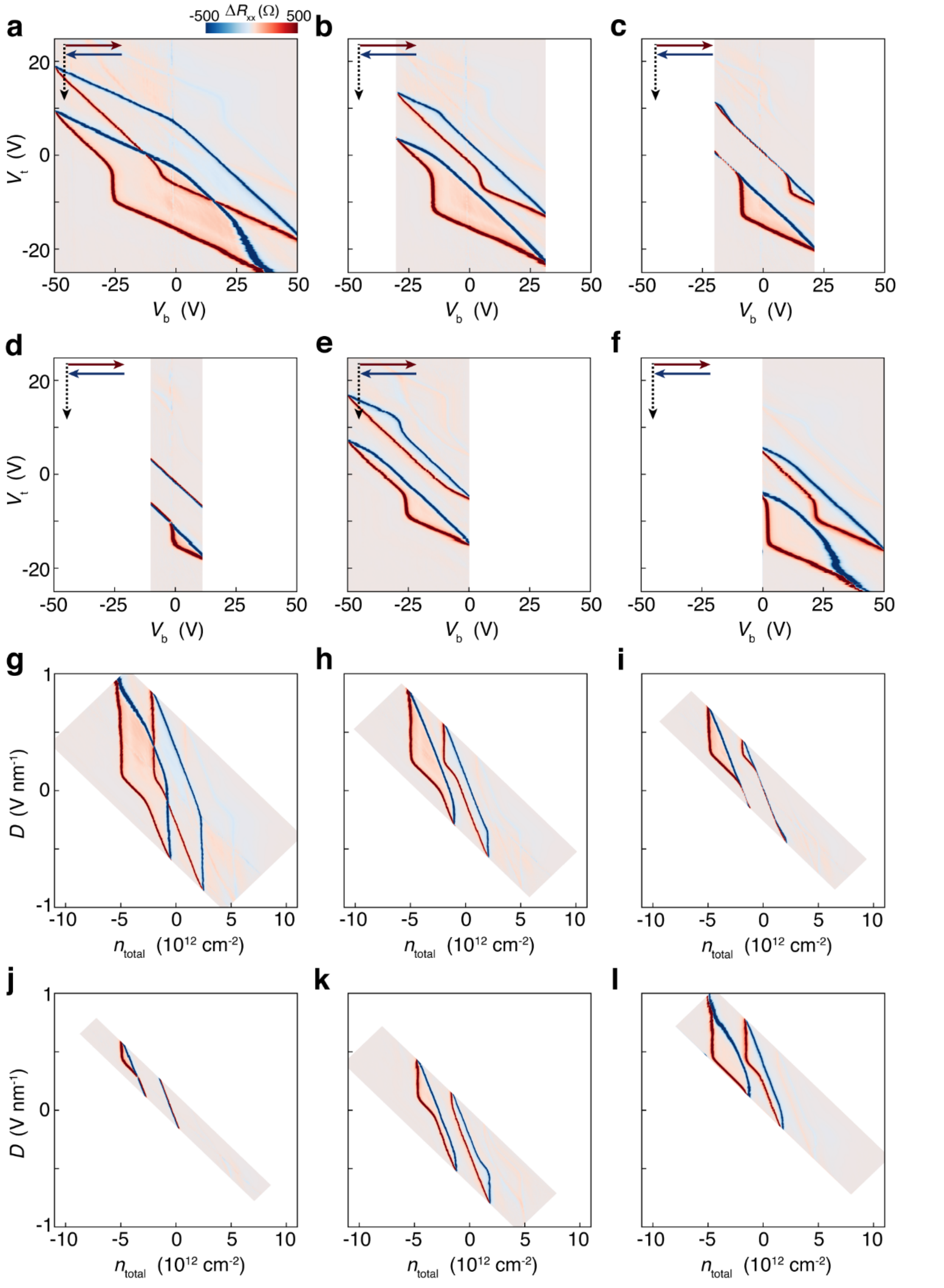


**Supplementary Fig. 10 | Dependence of ferroelectric hysteresis on the scan range of $V_\mathrm{b}$. a-f**, Difference in $R_{xx}$ between forward and backward scan as a function of $V_\mathrm{t}$ and $V_\mathrm{b}$. The solid (dashed) arrows illustrate the fast (slow) -scan direction. The scan ranges of $V_\mathrm{b}$ are ±50 V (**a**), ±30 V (**b**), ±20 V (**c**), ±10 V (**d**), -50 V to 0 V (**e**), and 0 V to 50 V (**f**). **g-l**, Replotted corresponding maps of resistance as a function of $n_\mathrm{total}$ and $D$. Data were acquired at 2.2 K and plotted in the same scale.

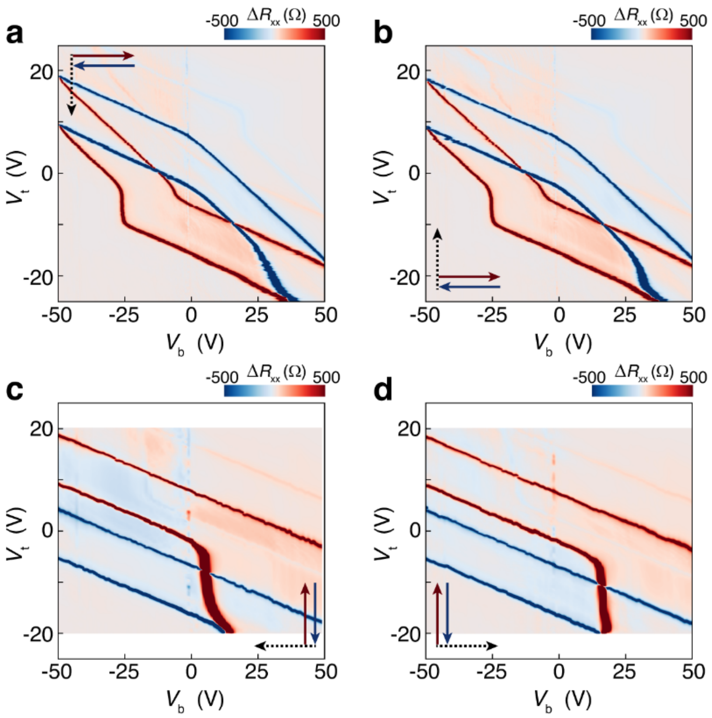


**Supplementary Fig. 11 | Independence of ferroelectric hysteresis of the slow-scan direction.** For better comparison, all the resistance difference are plotted as a function of $V_b$ ($x$-axis) and $V_t$ ($y$-axis). The slow-scan directions are marked as black dashed arrows, and the fast-scan directions are marked as red or blue solid arrows. **a**,**b**, Fast-scan axis ($V_b$) is swept between ±50 V forward and backward with slow-scan axis ($V_t$) decreasing from 25 V to -25 V (**a**), or increasing from -25 V to 25 V (**b**). **c**,**d**, Fast-scan axis ($V_t$) is swept between ±20 V forward and backward with slow-scan axis ($V_b$) decreasing from 50 V to -50 V (**c**), or increasing from -50 V to 50 V (**d**). The hysteresis is the same between **a** (**c**) and **b** (**d**), suggesting that the ferroelectric hysteresis is independent of the slow-scan direction.

## Supplementary Note 5: Detailed data from Hall measurements

Supplementary Fig. 12 presents how Hall resistance and the corresponding Hall carrier density evolve, depending on the scanning direction of electric field.

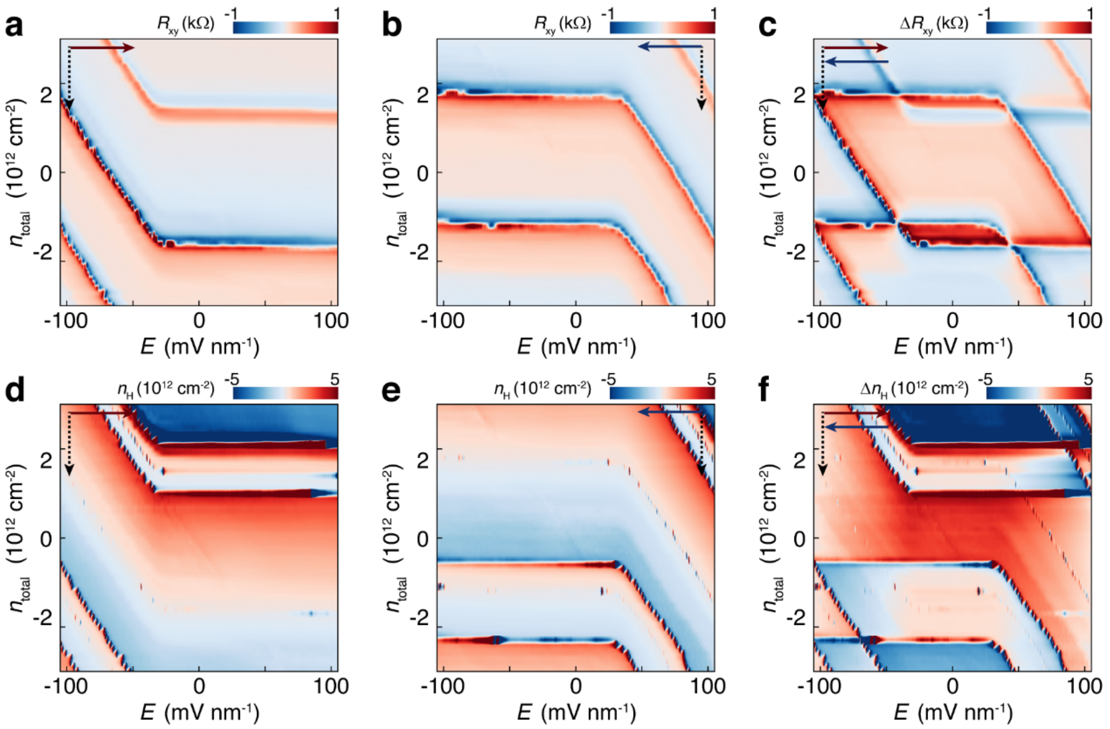


**Supplementary Fig. 12 | Hall measurements by scanning electric field. a**,**b**, The Hall resistance as a function of $n_{\text{total}}$ and $E$, measured by scanning electric field $E$ in the forward (**a**) and backward (**b**) directions at each fixed $n_{\text{total}}$ decreasing from $3\times10^{12}$ cm$^{-2}$ to $-3\times10^{12}$ cm$^{-2}$ with a step of $1\times10^{11}$ cm$^{-2}$. **c**, Difference of Hall resistance between (**a**) and (**b**). **d**,**e**, The corresponding Hall carrier density $n_{\text{H}} = -B/eR_{xy}$ of (**a**) and (**b**). **f**, Difference of Hall carrier density between (**d**) and (**e**). All the measurement was performed at $B = \pm 0.1$ T. The Hall resistance was anti-symmetrized to remove the component of longitudinal resistance caused by the imperfection of device configuration.

**Supplementary Note 6: Characterization of additional devices**

To figure out the layer and band structure dependent ferroelectricity in graphene moiré superlattices, we have made several controlled devices, summarized in Supplementary Table 1. Device D1-1, D1-2, and D1-3 share the same stack, but with monolayer, bilayer, and trilayer graphene, respectively. These devices allow us to *in-situ* compare the emergent ferroelectricity in graphene with various layer numbers, as already discussed in the main text.

Additionally, we made another device in which monolayer graphene is doubly aligned with both the top and bottom h-BN (see Supplementary Fig.13a). The ferroelectric hysteresis and GSAS can be reproduced in monolayer graphene/h-BN superlattice (Device D2-1) and twisted bilayer graphene aligned with h-BN (Device D2-2), as shown in Supplementary Fig.13 and 14, respectively. While the microscopic picture in the main text is described based on Device D1-1, the behavior in Device D2-1 can also be understood within the "asymmetric potential" framework. Although the monolayer graphene is aligned with both h-BN, the moiré potentials at top and bottom interface are different in terms of the moiré wavelength. As shown in Supplementary Fig.13c, there are two resistance peaks located at $V_{\text{b1}} = -15$ and $V_{\text{b2}} = -47$ V in the hole side, which correspond to two moiré wavelengths[12]. As mentioned in Supplementary Note 2, we can quantitatively determine the moiré wavelengths $\lambda_t = 13\,nm$, $\lambda_b = 8.6\,nm$ and the corresponding two twist angles $\theta_t = 0.58°$,

$\theta_b = 1.37°$ between graphene and top/bottom h-BN. Since the moiré potential is sensitive to the moiré wavelength, even slight difference in twist angles between graphene/top h-BN and graphene/bottom h-BN can significantly affect the moiré trapping ability, resulting in the asymmetric potential. With these results, we can further determine the relative angle between the top h-BN and bottom h-BN $\Theta_{BN}$ is either $1.95°$ or $0.79°$ as illustrated in Supplementary Fig.13f. Thus, the structure symmetry is absent in Device D2-1 as well.

Furthermore, the gate response between $V_t$ and $V_b$ are dramatic different in detail. In the dual-gate resistance mappings, we scan $V_t$ forward and backward between -20 V to 20 V at each fixed $V_b$. Intriguingly, $V_t$ is completely screened within the scanning range, resembling the behavior of the main device. We measured $R_{xx}$ by scanning $V_b$ at a fixed $V_t = 0$ V. Hysteresis behavior emerged when $|V_b|_{max} > 30$ V, identified by the non-overlapped resistance peaks of SDP on the hole side. Our data indicate that the top gate has stronger moiré trapping ability than bottom gate, exhibiting top gate anomalous screening effects. Similar behavior has been observed in bilayer graphene aligned with both top and bottom h-BN[1].

In twisted bilayer graphene device, we also observed the GSAS effect as the resistance trajectories of CNP and band insulators (BIs) are parallel to $V_t$. The ferroelectric hysteresis is dependent on the scanning range of $V_b$ as well. Regardless of the layer number and fine band structure of graphene, the electronic ferroelectricity is expected to be common to all graphene/$h$-BN superlattice with asymmetric moiré potential landscapes.

In the previous work and our current work, the unconventional ferroelectricity can only be observed in devices with top and bottom h-BN staggered by special angles[1,13]. We also made a control moiré device, in which the top and bottom h-BN are misaligned by 22°. Neither GSAS effect nor hysteresis was observed (Supplementary Fig.15). The underlying role of special configuration of top and bottom h-BN still remains to be understood.

**Supplementary Table 1: Summary of measured devices**.

| Device | layer | $\theta_t$ | $\theta_b$ | $\Theta_{BN}$ | top gate | bottom gate |
|---|---|---|---|---|---|---|
| D1-1 | monolayer | ± 0.68° | N/A | ~30° | screened & hystersis | partially screened & hystersis |
| D1-2 | bilayer | ± 0.68° | N/A | ~30° | screened & hystersis | partially screened & hystersis |
| D1-3 | trilayer | ± 0.68° | N/A | ~30° | screened & hystersis | partially screened & hystersis |
| D2-1 | monolayer | ± 0.58° | ± 1.37° | 1.95° or 0.79° | screened | hystersis |
| D2-2 | twisted bilayer | ± 0.58° | N/A | 1.95° or 0.79° | screened | partially screened & hystersis |
| D3 | monolayer | ± 0.57° | N/A | ~22° | normal gate | normal gate |

Note: $\theta_t/\theta_b$ is the twist angle between graphene and top/bottom h-BN determined by transport measurements. Due to the lack of measurable moiré transport features, we are not able to determine $\theta_b$ in these devices except D2-1. $\Theta_{BN}$ is the relative angle between the top h-BN and bottom h-BN determined by optical images and SHG measurements.

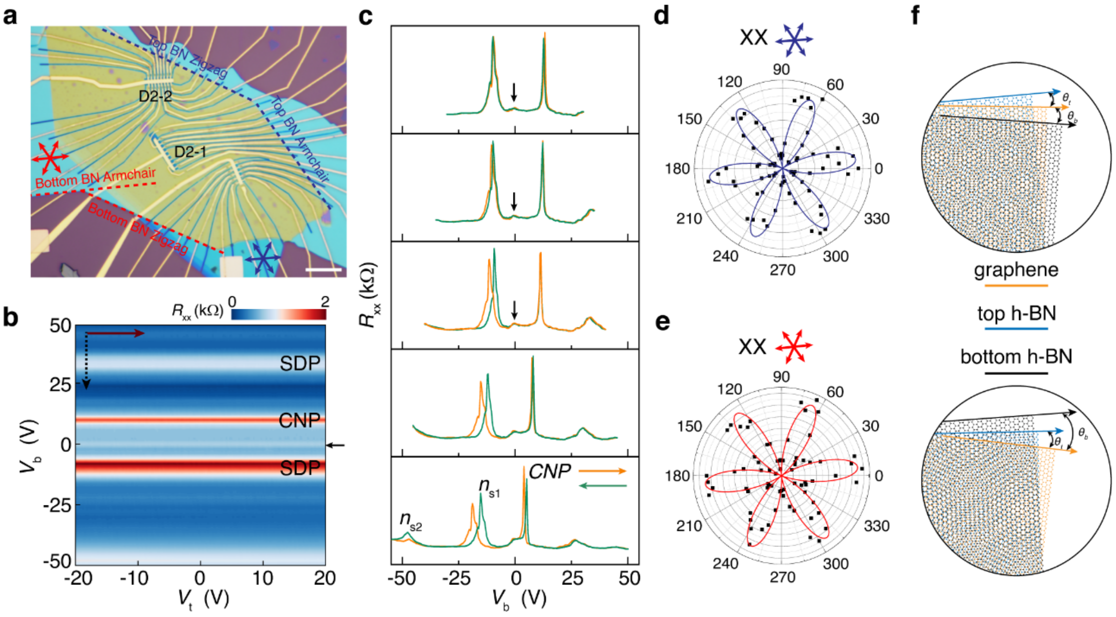


**Supplementary Fig. 13 | Ferroelectricity and GSAS in monolayer graphene superlattices for Device D2-1. a**, Optical image of Device D2. The bule (red) dash lines correspond to the armchair or zigzag crystallographic axes of top (bottom) h-BN. Device D2-1 is monolayer graphene/h-BN. Device D2-2 is twisted bilayer graphene aligned to h-BN. The scale bar is 10 μm. **b**, Dual-gate resistance maps as a function of $V_b$ (slow-scan axis) and $V_t$ (fast-scan axis). The top gate is completely screened within the scan range, resembling that of Supplementary Fig. 9d. **c**, Hysteresis dependence on scan range of $V_b$ at fixed $V_t = 0$. Since graphene is doubly aligned with h-BN, there is an additional moiré superlattice manifested by the resistance peaks between SDP and CNP, marked by black arrows in (**b**) and (**c**). **d**,**e**, SHG signals for bottom and top h-BN, respectively. The relative angle between them is ~0 degree. **f,** Two possible the lattice configuration illustrated with twist angles $\theta_t$ and $\theta_b$ between graphene and top/bottom h-BN. Both of which exhibit a nonzero twist angle between top h-BN and bottom h-BN.

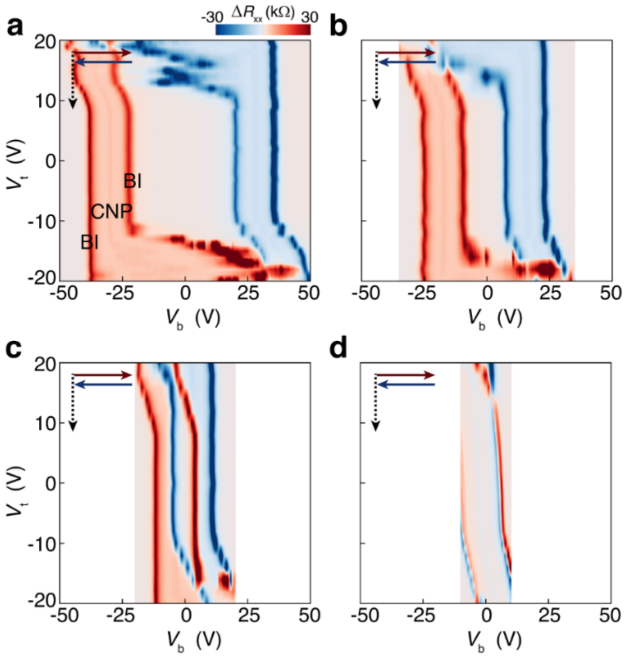


**Supplementary Fig. 14 | Ferroelectricity and GSAS in twisted bilayer graphene for Device D2-2. a**,**b**, Difference in $R_{xx}$ between forward and backward scan as a function of $V_\mathrm{t}$ and $V_\mathrm{b}$. The scanning range of $V_\mathrm{b}$ are ±50 V (**a**), ±30 V (**b**), ±20 V (**c**), ±10 V (**d**). The prominent resistance peaks correspond to the two band insulators (BI) of twisted bilayer graphene. There is relatively small resistance peak between the two BI, which is CNP. The data were acquired from Device D2-2 marked in Supplementary Fig. 13a.

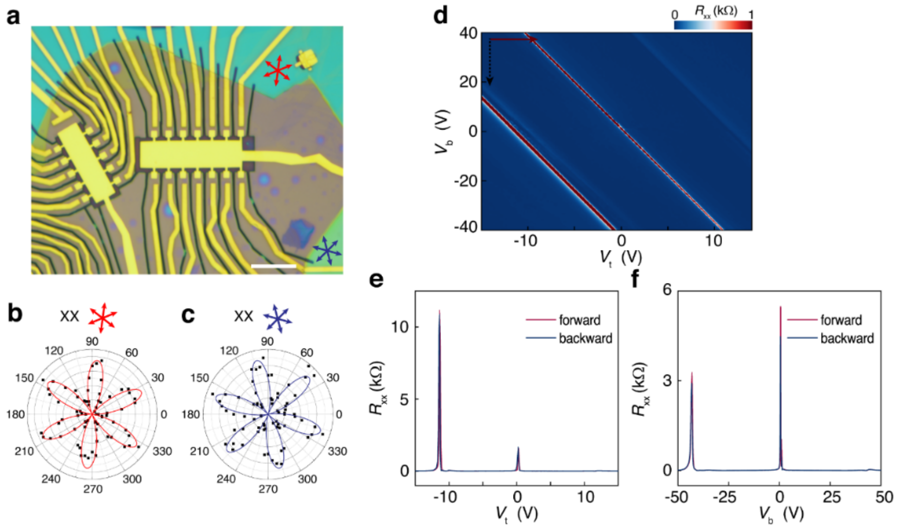


**Supplementary Fig. 15 | Absence of hysteresis and GSAS in a control monolayer graphene/h-BN moiré device. a,** Optical image of Device D3. The scale bar is 10 μm. **b**,**c**, SHG signals for bottom and top h-BN, respectively. The relative angle between them is ~20°. **d**, Dual-gate $R_{xx}$ maps as a function of $V_\mathrm{b}$ (slow-scan axis) and $V_\mathrm{t}$ (fast-scan axis). The resistance peaks of CNP and SDPs follow diagonal trajectories in the space of $V_\mathrm{b}$ and $V_\mathrm{t}$, indicating the absence of GSAS. **e**, $R_{xx}$ as a function of $V_\mathrm{t}$ by sweeping $V_\mathrm{t}$ forward and backward at $V_\mathrm{b} = 0$ V. **f**, $R_{xx}$ as a function of $V_\mathrm{b}$ by sweeping $V_\mathrm{b}$ forward and backward at $V_\mathrm{t} = 0$ V. Each forward scan curve is coincident with the backward scan one. Therefore, the ferroelectric hysteresis is absent in the control device.

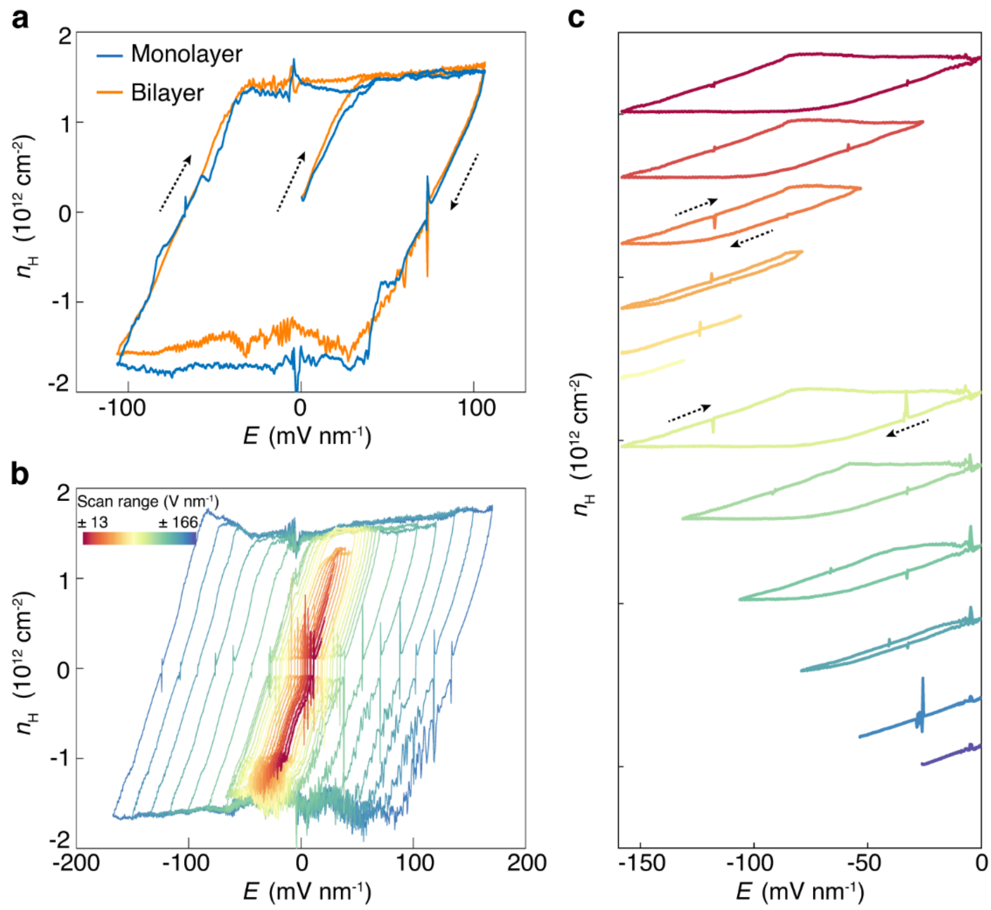


**Supplementary Fig. 16 | Ferroelectric loops in bilayer graphene. a**, Hall carrier density $n_{\mathrm{H}}$ as a function of $E$ in monolayer and bilayer graphene measured at $n_{\mathrm{total}} = 0$. The electric field was scanned in the direction of 0 → 106 → -106 → 106 mV nm$^{-1}$ marked as black dash arrows. **b**, Ferroelectric loops for various scanning ranges in bilayer graphene. All the loops are similar to those of monolayer shown in Fig. 3c. Particularly, the remanent Hall carrier density $n_{\mathrm{H}}^{\mathrm{s}}$ is the same as that in monolayer graphene, and approximately equal to $n_{\mathrm{Full}}/2$. The measured saturation itinerant density $n_{\mathrm{H}}^{\mathrm{s}} \approx 1.5 \times 10^{12}$ cm$^{-2}$ is independent of $|E|_{\mathrm{max}}$ and layer number, but highly relative to moiré period. In our device, the observation of SDP facilitates the determination of the full filling carrier density ($n_{\mathrm{Full}} = 3.0 \times 10^{12}$ cm$^{-2}$) of a moiré band. We found that the saturation occurs at half filling of the moiré band, as we have $n_{\mathrm{H}}^{\mathrm{s}} \approx 0.5 n_{\mathrm{Full}}$. Increasing temperature will thermally active the localized carriers, causing them to become itinerant ones. Therefore, we observed the decrease in $n_{\mathrm{H}}^{\mathrm{s}}$ (or $P_{\mathrm{s}}$) with increasing temperature, as shown in Fig. 4. **c**, Ferroelectric loops in arbitrary scanning ranges.

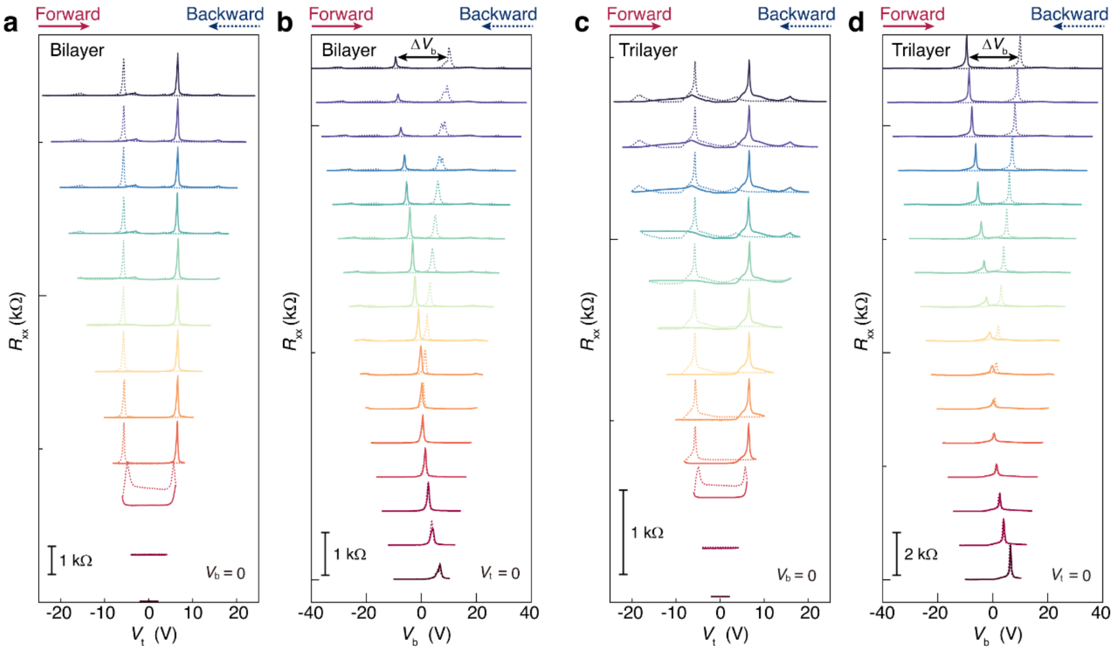


**Supplementary Fig. 17 | Ferroelectric hysteresis in bilayer and trilayer graphene. a**, $R_{xx}$ as a function of $V_t$ by sweeping $V_t$ in various ranges ($|V_t|_{max}$) while fixing $V_b = 0$. **b**, $R_{xx}$ as a function of $V_b$ by sweeping $V_b$ in various ranges ($|V_b|_{max}$) while fixing $V_t = 0$. The curves are vertically shift for clarity. (**a**) and (**b**) are acquired in bilayer graphene. **c**,**d**, Similar measurements to that in (**a**) and (**b**), but for trilayer graphene. Both bilayer (**a-b**) and tri-layer (**c-d**) graphene exhibit the same ferroelectric hysteresis as that in monolayer graphene presented in Fig. 1d-1e in the main text.

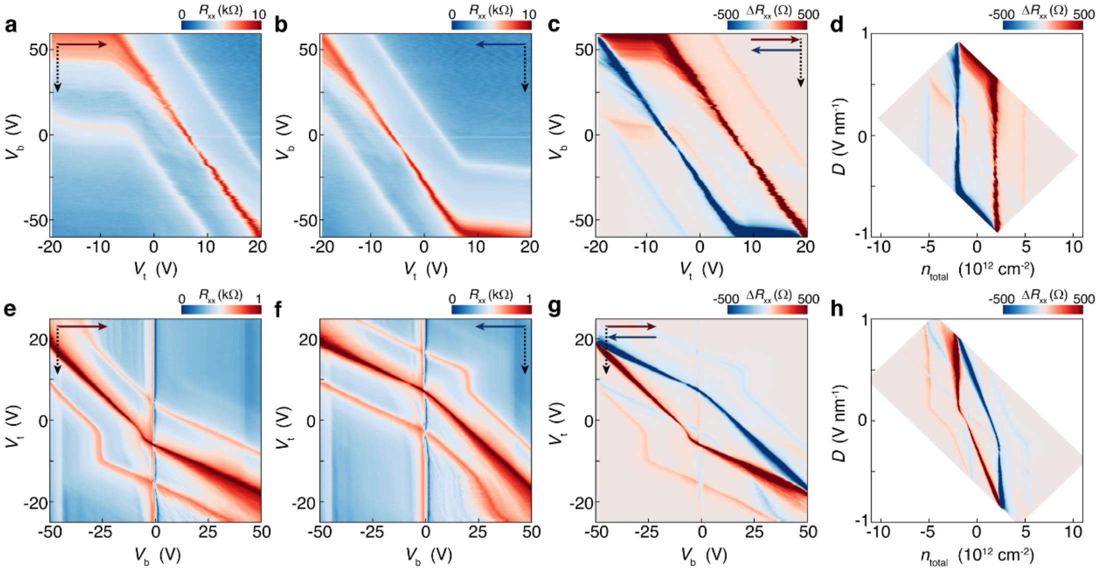


**Supplementary Fig. 18 | GSAS in bilayer graphene. a**,**b**, Dual-gate maps of $R_{xx}$ by scanning $V_t$ forward (**a**) and backward (**b**) at each fixed $V_b$. **c**, The difference between $R_{xx}$ in (**a**) and (**b**). **d**, The corresponding $n_{total} - D$ plot of (**c**). **e**,**f**, Dual-gate maps of $R_{xx}$ by scanning $V_b$ forward (**e**) and backward (**f**) at each fixed $V_t$. **g**, The difference between $R_{xx}$ in (**e**) and (**f**). **h**, The corresponding $n_{total} - D$ plot of (**g**). The data is collected simultaneously with that of Fig. 2 in the main text. The GSAS behavior in bilayer moiré structure is identical to that in monolayer counterpart.

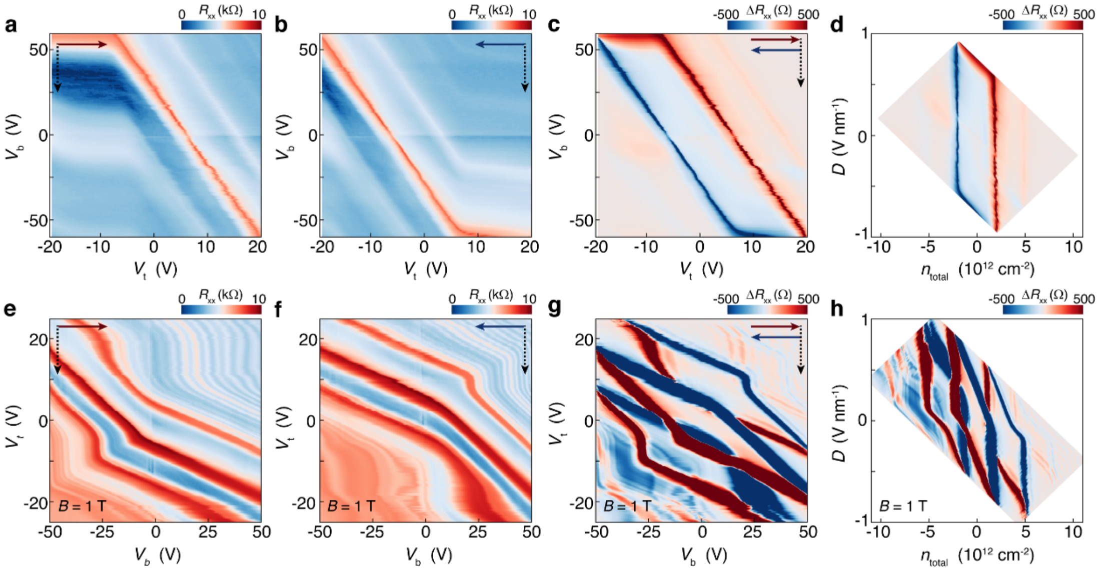


**Supplementary Fig. 19 | GSAS in trilayer graphene. a**,**b**, Dual-gate maps of $R_{xx}$ by scanning $V_t$ forward (**a**) and backward (**b**) at each fixed $V_b$ without magnetic field. **c**, The difference between $R_{xx}$ in (**a**) and (**b**). **d**, The corresponding $n_{total} - D$ plot of (**c**). **e**,**f**, Dual-gate maps of $R_{xx}$ by scanning $V_b$ forward (**e**) and backward (**f**) at each fixed $V_t$ with magnetic field $B = 1$ T. **g**, The difference between $R_{xx}$ in (**e**) and (**f**). **h**, The corresponding $n_{total} - D$ plot of (**g**). The electronic states of Landau levels also exhibit screening and hysteresis behaviors.

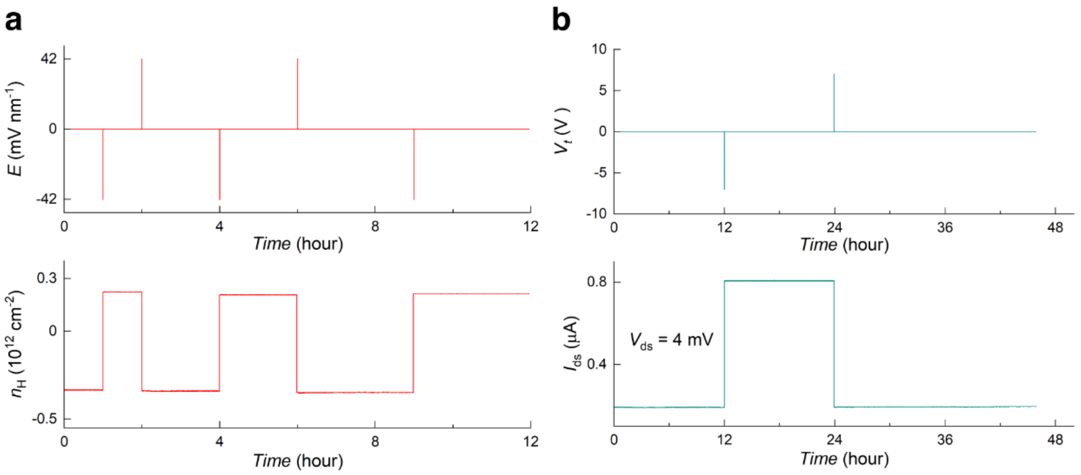


**Supplementary Fig. 20 | Stability of polarization states. a,** $n_{\mathrm{H}}$ measured after the positive pulse of $E$ =42 mV nm$^{-1}$ and negative pulse of $E$ =-42 mV nm$^{-1}$for 1, 2 and 3 hours. Neither state shows degradation over a relevant retention time. **b**, $I_{\mathrm{ds}}$ measured with constant source-drain voltage $V_{\mathrm{ds}} = 4$ mV after the positive pulse of $V_t$ = 7 V and negative pulse of $V_t$ = -7 V for 12 and 24 hours. Neither state shows degradation over the long retention time.

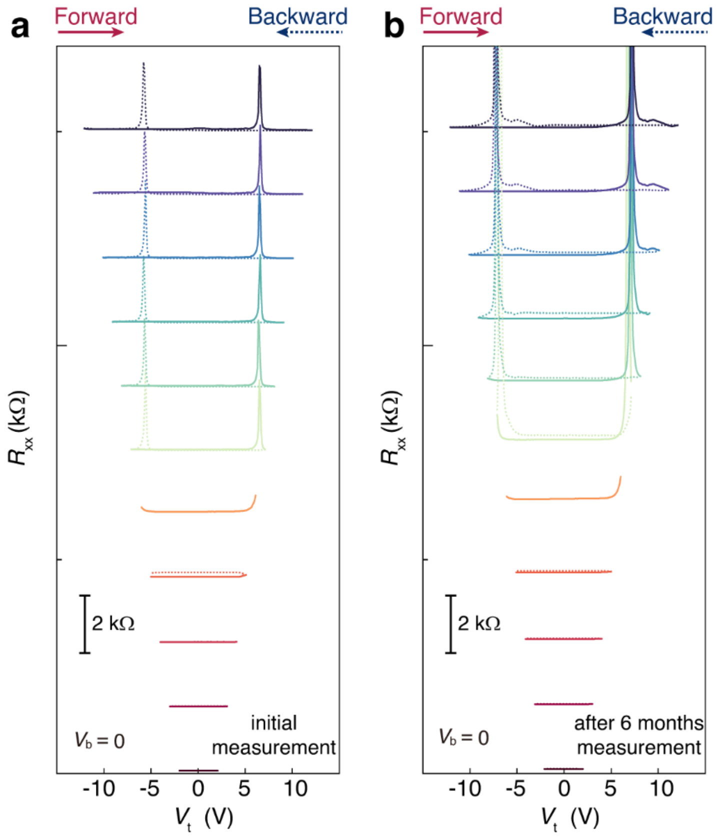


**Supplementary Fig. 21 | Stability test.** Initial (**a**) and repeated (**b**) measurements of $R_{xx}$ versus $V_{\mathrm{t}}$ by sweeping $V_{\mathrm{t}}$ in various ranges ($|V_{\mathrm{t}}|_{\mathrm{max}}$) while fixing $V_{\mathrm{b}} = 0$. The scanning range $|V_{\mathrm{t}}|_{\mathrm{max}}$ increases from 2 V to 12 V with interval of 1 V. The curves in (**a**) and (**b**) are vertically shifted for clarity. The forward and backward sweeps are shown in solid and dashed lines, respectively.

## Supplementary References:


1 Zheng, Z. *et al.* Unconventional ferroelectricity in moire heterostructures. *Nature* **588**, 71-76 (2020).

2 Zheng, Z. *et al.* Electronic ratchet effect in a moiré system: signatures of excitonic ferroelectricity. *arXiv*, arXiv:2306.03922 (2023).

3 Ferrari, A. C. & Basko, D. M. Raman spectroscopy as a versatile tool for studying the properties of graphene. *Nat. Nanotechnol.* **8**, 235-246 (2013).

4 Finney, N. R. *et al.* Tunable crystal symmetry in graphene-boron nitride heterostructures with coexisting moire superlattices. *Nat. Nanotechnol.* **14**, 1029-1034 (2019).

5 Hunt, B. *et al.* Massive Dirac fermions and Hofstadter butterfly in a van der Waals heterostructure. *Science* **340**, 1427-1430 (2013).

6 Wang, L. *et al.* Evidence for a fractional fractal quantum Hall effect in graphene superlattices. *Science* **350**, 1231-1234 (2015).

7 Ponomarenko, L. A. *et al.* Cloning of Dirac fermions in graphene superlattices. *Nature* **497**, 594-597 (2013).

8 Berdyugin, A. I. *et al.* Out-of-equilibrium criticalities in graphene superlattices. *Science* **375**, 430-433 (2022).

9 Dean, C. R. *et al.* Hofstadter's butterfly and the fractal quantum Hall effect in moiré superlattices. *Nature* **497**, 598-602 (2013).

10 Li, C. *et al.* Signature of gate-tunable magnetism in graphene grafted with Pt-porphyrins. *Physical Review B* **93**, 045403 (2016).

11 Wang, H., Wu, Y., Cong, C., Shang, J. & Yu, T. Hysteresis of electronic transport in graphene transistors. *ACS Nano* **4**, 7221-7228 (2010).

12 Wang, Z. *et al.* Composite super-moire lattices in double-aligned graphene heterostructures. *Sci Adv* **5**, eaay8897 (2019).

13 Niu, R. *et al.* Giant ferroelectric polarization in a bilayer graphene heterostructure. *Nat. Commun.* **13**, 6241 (2022).
